# Aggregation Behavior of Long-Chain Piperidinium Ionic Liquids in Ethylammonium Nitrate

**DOI:** 10.3390/molecules191220157

**Published:** 2014-12-02

**Authors:** Caili Dai, Mingyong Du, Yifei Liu, Shilu Wang, Jianhui Zhao, Ang Chen, Dongxu Peng, Mingwei Zhao

**Affiliations:** State Key Laboratory of Heavy Oil Processing, School of Petroleum Engineering, China University of Petroleum (Huadong), Qingdao 266580, Shandong, China; E-Mails: daicl306@163.com (C.D.); upcdmy@163.com (M.D.); zgsydxliuyifei@163.com (Y.L.); wangshilu2009@126.com (S.W.); jessica_zjh@hotmail.com (J.Z.); chenang1016@126.com (A.C.); pengdx731@163.com (D.P.)

**Keywords:** aggregation behavior, long-chain piperidinium ionic liquid, surface tension, dissipative particle dynamics

## Abstract

Micelles formed by the long-chain piperidinium ionic liquids (ILs) *N*-alkyl-*N*-methylpiperidinium bromide of general formula C_n_PDB (*n* = 12, 14, 16) in ethylammonium nitrate (EAN) were investigated through surface tension and dissipative particle dynamics (DPD) simulations. Through surface tension measurements, the critical micelle concentration (*cmc*), the effectiveness of surface tension reduction (Π*_cmc_*), the maximum excess surface concentration (Г*_max_*) and the minimum area occupied per surfactant molecule (*A_min_*) can be obtained. A series of thermodynamic parameters ($\Delta G_{\text{m}}^{0}$, $\Delta H_{\text{m}}^{0}$ and $\Delta S_{\text{m}}^{0}$) of micellization can be calculated and the results showed that the micellization was entropy-driven. In addition, the DPD simulation was performed to simulate the whole aggregation process behavior to better reveal the micelle formation process.

## 1. Introduction

Ionic liquids (ILs) are a class of organic salts that are liquids at or near room temperature. They have attracted much attention because of their special properties, such as low volatility, nonflammability, high thermal stability and high ionic conductivity [1,2,3,4,5,6,7,8,9,10,11,12]. These characteristics make ILs attractive alternatives to traditional organic solvents [13,14,15]. There is now extensive literature reporting the successive synthesis and investigation of a large number of ILs. ILs are based on imidazolium, pyrrolidinium, pyridinium, piperidinium and quaternary ammonium cations. The anions may vary, for example, halides, PF_6_^−^, BF_4_^−^, (CF_3_SO_3_)_2_N^−^ and CF_3_SO_3_^−^. These materials are widely used in organic synthesis, catalysis and preparation of nanostructured matters [16,17,18,19]. ILs with long alkyl chains can be regarded as a novel kind of amphiphilic molecule. In recent years, numerous papers have reported the aggregation behavior of IL-type surfactants in aqueous solution [20,21,22,23,24,25,26]. In this context, piperidinium-based ILs have been investigated recently [27,28,29,30]. The Chen group studied the phase behavior of a series of piperidinium ILs using Polarized Optical Microscopy (POM), Small-Angle X-Ray Scattering (SAXS) and rheology measurements [31]. Milioto and his co-workers investigated the thermodynamic properties of a series of long-chain piperidinium salts in water [32]. Zhao *et al.*, investigated the micelle behavior of piperidinium ILs *N*-alkyl-*N*-methylpiperidinium bromide C_n_PDB (*n* = 12, 14, 16) through surface tension, electrical conductivity and steady-state fluorescence measurements [33].

Ethylammonium nitrate (EAN) is a room-temperature ionic liquid (RTIL) discovered in 1914 [34]. EAN has been widely investigated and used in many fields. Dielectric spectroscopy studies were carried out in order to study the dielectric behavior of EAN [35]. In protein chemistry, EAN has many potential applications, for example, it can be used as an additive, a detergent, a precipitating agent or to deliver ligands to protein crystals [36,37]. Phase behaviors of surfactants and lipids in EAN were studied over 20 years ago [38,39,40,41,42,43]. EAN is a protic ionic liquid and has the ability to form a three-dimensional hydrogen-bond network, which is a characteristic supporting self-assembly of a surfactant [44]. Zheng group studied the aggregation behavior of some 1-alkyl-*3*-methylimidazolium bromides (C_n_mimBr, *n* = 12, 14, 16) in EAN. They concluded that C_n_mimBr can form micelles in EAN, then investigated the solvophobic interactions between the hydrocarbon chains of C_n_mimBr and EAN molecules [45]. The aggregation behavior and micelle formation mechanism of *N*-alkyl-*N*-methylpyrrolidinium bromide (C_n_MPB *n* = 12, 14, 16) in EAN were investigated through surface tension measurement and ^1^H-NMR spectrometry by Shi and coworkers [46]. The Drummond group has studied the self-assembly of hexadecyltrimethylammonium bromide (CTAB), myverol 18–99 K and phytantriol in many protic ILs, including EAN [47,48]. Recently, research has focused on the aggregation behavior of surface active ILs in RTIL, including in EAN [49,50].

In the present work, we prepared a series of piperidinium ILs with different alkyl chain lengths, C_n_PDB (*n* = 12, 14, 16). The aggregation behaviors of these ILs in EAN have been investigated by surface tension measurements and dissipative particle dynamics (DPD) simulations. Our aim was to examine the influence of alkyl chain length on the aggregation behavior, so that we can offer a systematic study of the mechanism of formation of aggregations formed by surface active ILs in RTILs.

## 2. Results and Discussion

### 2.1. Surface Tension of C_n_PDB in EAN

**Figure 1:**
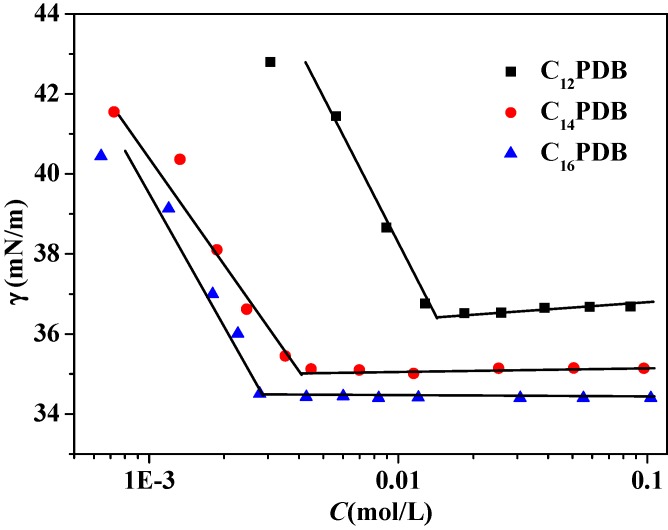
Surface tension as a function of C_n_PDB concentration at 298.15 K.

Figure 1 shows the surface tension of C_n_PDB (*n* = 12, 14, 16) in EAN at various concentrations at 298 K. The surface tension of the C_n_PDB solution decreases sharply at the beginning compared with pure EAN. As C_n_PDB concentrations increase further, the surface tension decreases gradually. Finally, the surface tension remains constant above the critical micelle concentration (*cmc*). The *cmc* values are listed in Table 1. The *cmc* value of a surfactant reflects its surface properties, a smaller *cmc* value means better surface activity. The value of *cmc* declines with the increase of hydrocarbon chain length, which is similar to their aggregation behavior in aqueous solution. The result suggests that there exist solvophobic interactions between the hydrocarbon chain and EAN, similar to the hydrophobic interactions in water. The obtained *cmc* values of C_n_PDB are higher than those in aqueous solution [32]. The *cmc* values of C_n_PDBs in EAN are smaller than those of C_n_mimBr (0.139, 0.0350 and 0.00913 mol·L^−1^) and C_n_MPB (0.097, 0.026 and 0.0078 mol·L^−1^) for the same alkyl chain length [45,46]. The cations of C_n_mimBr and C_n_MPB have a great impact on that. There are two main two reasons, head groups have opposing tendencies to keep close to minimize hydrocarbon-solvent contacts and to repel as a result of electrostatic repulsion, solvation and steric hindrance [51]. The reason might be the lower hydrophilicity of the C_n_PDB head groups than that of C_n_mimBr and C_n_MPB.

**Table 1:** Surface properties of C_n_PDB (*n* = 12, 14, 16) in EAN at 298.15K.

ILs	*cmc* (×10^3^ mol/L)	*γ_cmc_* (mN/m)	Π*_cmc_* (mN/m)	Г*_max_* (μmol/m^2^)	*A_min_* (Å^2^)
C_12_PDB	13.7 ± 0.5	36.412 ± 0.001	12.935 ± 0.001	0.372	446.5
C_14_PDB	4.7 ± 0.03	35.112 ± 0.001	14.351 ± 0.001	1.201	138.3
C_16_PDB	2.8 ± 0.03	34.610 ± 0.001	14.737 ± 0.001	1.390	119.4

**Figure 2:**
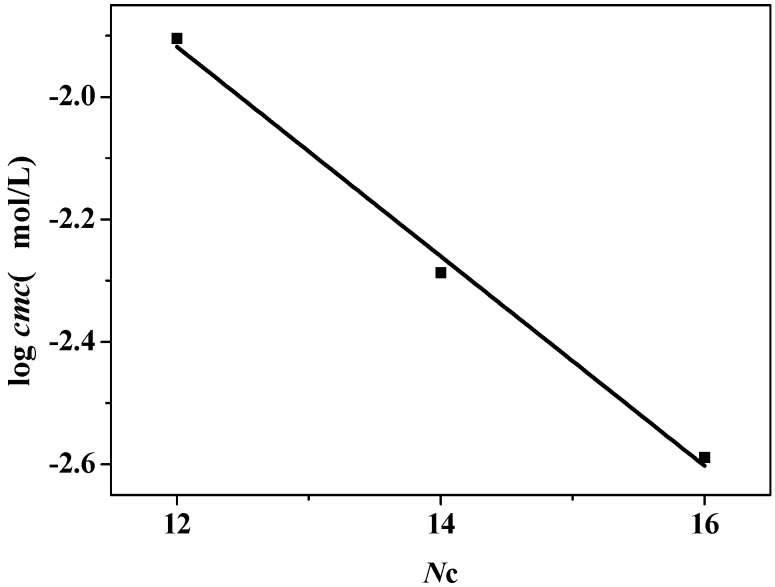
Plot of logarithmic *cmc* *versus* the number of carbon atoms in the hydrocarbon chain of C_n_PDB at 298.15 K.

Figure 2 shows the relationship between the number of carbon atoms in the hydrocarbon chain of C_n_PDB and lg*cmc*. Figure 2 shows that lg*cmc* decreases with the increase of alkyl chain length and the plot is almost liner. The rule can be expressed by the empirical formula:

(1) lg*cmc* = A − BNc

In this formula, A and B are constants. A stands for the ability of forming micelles of a surfactant and B stands for the average contribution to the micelle formation by the methylene in the hydrophobic chain. The value of A is obtained by extrapolation of the straight line and A and B were calculated to be 0.1807 and 0.1736, respectively. The value of B for C_n_PDB is similar with C_n_MPB (0.28) and C_n_mimBr (0.30). The value of A for C_n_MPB and C_n_mimBr in EAN are 2.25, and 2.10, respectively. The lower value of A for C_n_PDB indicates that C_n_PDB is easier to form micelle in EAN, which is in accordance with the result from the comparison of surface tension. This phenomenon is resulted from the special interactions between the different head groups and EAN.

The effectiveness of surface tension reduction (Π*_cmc_*) can be obtained using the following formula:

(2) Π*_cmc_* = γ*_0_* − γ*_cmc_*

where γ_0_ is the surface tension of pure solvent and γ*_cmc_* is the surface tension of the solvent. The values are listed in Table 1. The results indicate that Π*_cmc_* decreases with the increase of the length of hydrocarbon chain, and when *n* = 16, the IL behaves best in reducing the surface tension, which indicates the ILs with longer hydrocarbon chains can reduce surface tension easier.

The maximum excess surface concentration (Г*_max_*) and the minimum area occupied per surfactant molecule (*A_min_*) at the air/liquid surface can be obtained from the Gibbs adsorption isotherm:

(3) $${\text{Γ}}_{max}=-\frac{1}{nRT}{\left(\frac{d\gamma }{d\text{ln}C}\right)}_{T}$$

(4) $$A_{min}=\frac{1}{N_{A}\Gamma_{max}}$$

where *R* is the gas constant (8.314 J·mol^−1^·K^−1^), T is the absolute temperature and the value of *n* is taken as 2 [52], *d*γ/*d*(ln*C*) is the slope of γ versus ln*C* dependence while the concentration is near *cmc*, *N_A_* is Avogadro’s number (6.022 × 10^23^ mol^−1^).

The value of Г*_max_* and *A_min_* obtained from the Gibbs adsorption isotherm reflects the molecule arrangement of ILs at the air/liquid interface [53] and they are listed in Table 1. With the increase of alkyl chain length, Г*_max_* increases but *A_min_* decreases, which means the longer alkyl chain can make the C_n_PDB molecules packing more closely. Compared with the values of C_n_PDB with the same alkyl chain length in water, Г*_max_* is larger but *A_min_* is smaller in EAN than that of in water. [31] This indicate that less C_n_PDB molecules would aggregate in air/EAN interface. The estimated Г*_max_* values for C_n_MPB in EAN are 0.96, 1.36 and 1.85 μmol/m^2^, the *A_min_* of that are 173, 122 and 89 Å. This means C_n_MPB molecules have a higher packing density at the air/EAN interface.

### 2.2. Temperature Dependence of cmc

**Figure 3:**
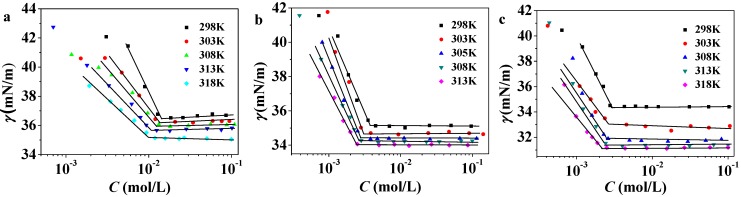
Surface tensions *versus* concentration at different temperatures of C_12_PDB (**a**); C_14_PDB (**b**); C_16_PDB (**c**) in EAN.

**Figure 4:**
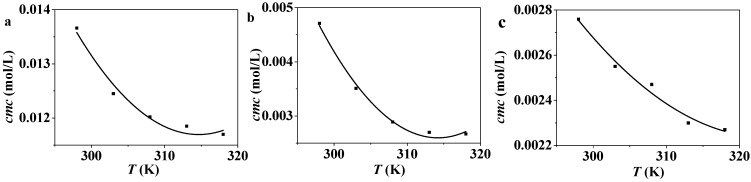
Plots of *cmc* *versus* temperature of C_12_MDB (**a**), C_14_MDB (**b**), C_16_MDB (**c**).

Plots of surface tensions against C_n_PDB concentrations at various temperatures are shown in Figure 3. The values of *cmc* for C_n_PDB at various temperatures are listed in Table 2. Figure 4 gives the correlations between *cmc* and temperature. It indicates that the value of *cmc* decreases with the temperature increase in a trend of U-shape and fits with a second-order polynomial. This trend is similar to the other ILs in EAN [46,47].

**Table 2:** Critical micelle concentration (*cmc*) and thermodynamic parameters of micellization for C_n_PDB (*n* =12, 14, 16) in EAN at various temperatures.

ILs	T (K)	*cmc* (×10^3^ mol·L)	$\Delta G_{m}^{0}$ (kJ·mol^−1^)	$\text{∆}H_{m}^{0}$ (kJ·mol^−1^)	$-T\text{∆}S_{m}^{0}$ (kJ·mol^−1^)
C_12_PDB	298	13.7 ± 0.05	−21.77 ± 0.009	10.73 ± 0.161	−32.50 ± 0.152
	303	12.5 ± 0.06	−22.30 ± 0.012	9.172 ± 0.106	−31.47 ± 0.094
	308	12.0 ± 0.03	−22.83 ± 0.006	7.667 ± 0.053	−30.49 ± 0.046
	313	11.9 ± 0.02	−23.28 ± 0.004	6.210 ± 0.001	−29.49 ± 0.003
	318	11.7 ± 0.04	−23.76 ± 0.009	4.799 ± 0.049	−28.56 ± 0.058
C_14_PDB	298	4.7 ± 0.03	−24.49 ± 0.017	52.94 ± 0.590	−77.43 ± 0.607
	303	3.5 ± 0.02	−25.89 ± 0.016	36.47 ± 0.296	−62.36 ± 0.312
	308	2.9 ± 0.02	−26.48 ± 0.018	20.54 ± 0.011	−47.02 ± 0.028
	313	2.7 ± 0.04	−27.24 ± 0.037	5.109 ± 0.265	−32.35 ± 0.228
	318	2.6 ± 0.01	−27.68 ± 0.010	−9.83 ± 0.532	−17.85 ± 0.522
C_16_PDB	298	2.8 ± 0.03	−25.66 ± 0.027	15.47 ± 0.970	−41.13 ± 0.943
	303	2.6 ± 0.02	−26.36 ± 0.020	12.63 ± 0.417	−38.99 ± 0.397
	308	2.5 ± 0.01	−26.99 ± 0.010	9.887 ± 0.119	−36.87 ± 0.129
	313	2.3 ± 0.02	−27.49 ± 0.023	7.231 ± 0.637	−34.72 ± 0.660
	318	2.2 ± 0.02	−28.09 ± 0.035	4.658 ± 0.139	−32.75 ± 0.174

### 2.3. Thermodynamic Analysis on the Micelle Formation of C_n_PDB in EAN

As is shown in Figure 4, the temperature has a significant relationship with the micelle formation of C_n_PDB in EAN. The standard Gibbs free energy of micelle formation is given as follows:

(5) $$\Delta G_{m}^{0}=2RT\text{ln}X_{S}$$

where $\Delta G_{m}^{0}$ is the standard Gibbs free energy; *R* is the gas constant; *T* is the absolute temperature; *X_S_* is the mole fraction of surfactant monomer coexisting with the micelle.

Then, the enthalpy of aggregation formation can be calculated by the Gibbs-Helmholtz Equation:

(6) $$\text{∆}H_{m}^{0}=\left[\frac{\partial \left(\frac{\text{∆}G_{m}^{0}}{T}\right)}{\partial \left(\frac{1}{T}\right)}\right]$$

On the basis of $\Delta G_{m}^{0}$ and $\text{∆}H_{m}^{0}$, $\text{∆}S_{m}^{0}$ can be derived as the following equation:

(7) $$\text{∆}S_{m}^{0}=\frac{\text{∆}H_{m}^{0}-\text{∆}G_{m}^{0}}{T}$$

**Figure 5:**
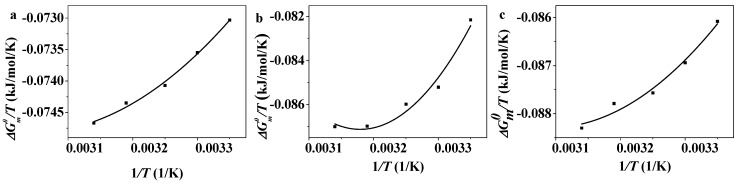
Plots of $\Delta G_{m}^{0}/T$ against *1*/*T* of C_12_PDB (**a**); C_14_PDB (**b**); and C_16_PDB (**c**).

**Figure 6:**
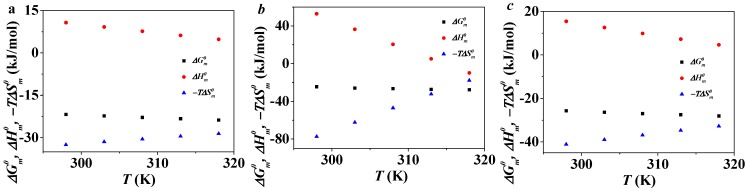
Plots of $\Delta G_{m}^{0}$, $\text{∆}H_{m}^{0}$ and $-T\text{∆}S_{m}^{0}$ *versus* *T* for C_12_MDB (**a**); C_14_MDB (**b**); and C_16_MDB (**c**).

The value of $\Delta G_{m}^{0}$ at different temperatures can be calculated based on Equation (5). As is shown in Figure 5, the value of $\Delta G_{m}^{0}/T$ increases along with the increase of 1/T. The plot fits with a second-order polynomial and the quadratic equations can be obtained. The values of $\text{∆}H_{m}^{0}$ and $-T\text{∆}S_{m}^{0}$ of C_12_PDB, C_14_PDB and C_16_PDB at different temperatures can be calculated according to Equations (6) and (7). Figure 6 shows the plots of $\text{∆}G_{m}^{0}$, $\text{∆}H_{m}^{0}$ and $-T\text{∆}S_{m}^{0}$ *versus* temperature of C_n_PDB (*n* = 12, 14, 16). $\Delta G_{m}^{0}$ is negative and decreases with the increase of temperature which is similar to the other ILs in EAN. From 298 K to 318 K, $-T\text{∆}S_{m}^{0}$ increases with the temperature while the value of $\text{∆}H_{m}^{0}$ decreases. The figure indicates that the negative $\Delta G_{m}^{0}$ is mainly contributed by the large negative $-T\text{∆}S_{m}^{0}$. Thus, the micelle formation of C_n_PDB (*n* = 12, 14, 16) in EAN is an entropy-driven process.

### 2.4. Dissipative Particle Dynamics (DPD) Simulation on the Micelle Formation of C_n_PDB in EAN

**Figure 7:**
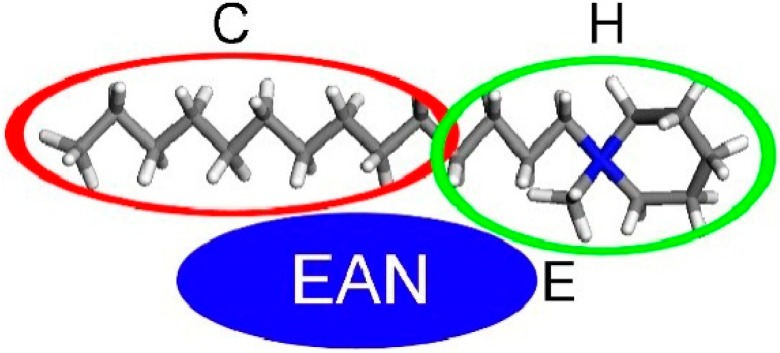
Simulation model of C_n_PDB in EAN. The C_n_PDB molecule is divided into two parts, alkyl-chain (**C**) and headgroup (**H**). Water is represented by (**E**).

The DPD simulation was performed using the Material Studio software. The theory of this simulation method has been discussed previously [31,54]. In the simulation model, the C_n_PDB molecule is shown in Figure 7 and the amphiphilic molecule is divided into two parts, the hydrophilic part C and the hydrophobic part H, which are connected by a harmonic spring and the monomer particle E represents for EAN. The model is simulated in a 10 × 10 × 10 cubic box. The temperature is kept at 298 K and the step size of the Newton equation for the integration is set to *Δt* = 0.05.

**Figure 8:**
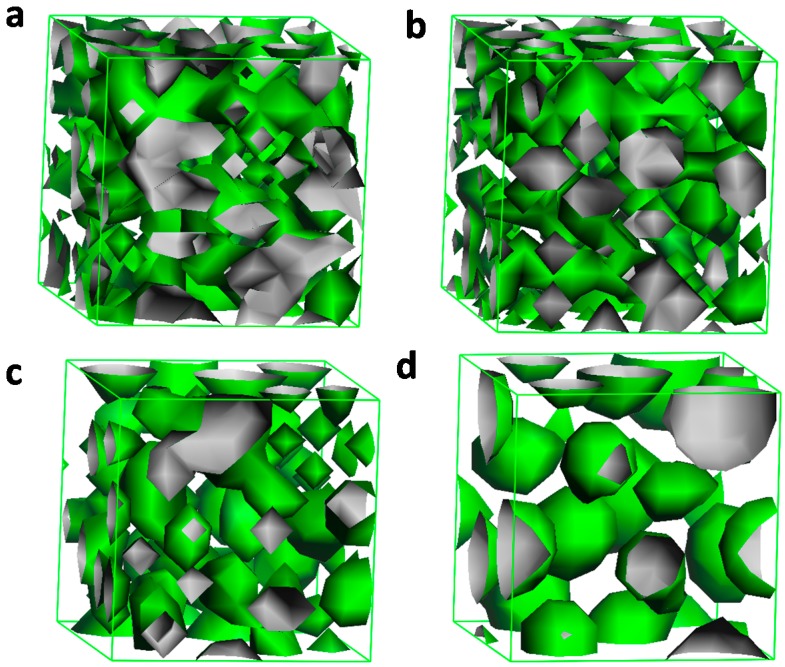
Simulation of micelle formation of 20% C_n_PDB in EAN at room temperature at different time steps: (**a**) 3; (**b**) 10; (**c**) 100; (**d**) 20,000. The size of the simulation model is 10 × 10 × 10 in DPD units.

In order to represent dynamic process for the micelle formation, 20% C_n_PDB is used to perform the DPD simulation and the results are shown in Figure 8. At first, the system is unstable and the beads are unordered, which can be seen from Figure 8a,b. No ordered structure is formed in this step. Then, Figure 8c indicates that some spherical structures are formed, but not very regular. At last, an ordered structure is finally formed and the structure is more ordered (Figure 8d). The process often happens in tens of μs and is difficult to observe in a lab experiment, so the simulated result is regarded as an effective method supplying valuable information about microphase separation.

## 3. Experimental Section

### 3.1. Materials

The compounds 1-methylpiperidinium (97%), 1-bromododecane (97%), 1-bromotetradecane (97%), 1-bromohexadecane (97%), ethylamine, nitric acid (65%), ethyl ether, 2-butanone (99%), and tetrahydrofuran (THF) were purchased from Aladdin Chemical Reagent Co., Ltd. (Shanghai, China)

#### 3.1.1. Synthesis of C_n_PDB (*n* = 12, 14, 16)

C_n_PDBs were synthesized according to a previously reported procedure [55]. A solution of 1-bromoalkane in 2-butanone was added dropwise to a solution of *N*-methylpiperidine in 2-butanone. The mixture was refluxed at 75–80 °C under a nitrogen atmosphere for 48 h. After cooling to room temperature, the 2-butanone was evaporated and the product was recrystallized from fresh tetrahydrofuran THF at least three times. Then it was dried under vacuum for 48 h at 50 °C. The products were characterized by ^1^H-NMR spectroscopy (400 MHz) using CDCl_3_ as solvent. The ^1^H-NMR data were recorded as follows:

C_12_PDB δ_H_: 0.881 (t, 3 H), 1.255–1.368 (m, 18 H), 1.727–1.940 (8 H), 3.346 (s, 3 H), 3.615–3.692 (m, 4 H), 3.775–3.806 (m, 2 H).

C_14_PDB δ_H_: 0.882 (t, 3 H), 1.255–1.369 (m, 22 H), 1.696–1.934 (m, 8 H), 3.367 (s, 3 H), 3.617–3.660 (m, 4 H), 3.821–3.833 (m, 2 H).

C_16_PDB δ_H_: 0.881 (t, 3 H), 1.255–1.368 (m, 26 H), 1.714–1.907 (m, 8 H), 3.362 (s, 3 H), 3.612–3.674 (m, 4 H), 3.824–3.851 (m, 2 H).

#### 3.1.2. Synthesis of EAN

EAN was synthesized according to Evans *et al.* [44]. A portion of nitric acid was added dropwise to ethylamine solution under stirring and cooling in an ice bath. Then water was removed from the resulting product with a rotary evaporator. EAN was identified by its ^1^H-NMR spectrum as follows: δ_H_ D_3_-AN use common abbreviation): 1.14 (t, 3H), 2.84 (m, 2H), 7.07 (s, 3H).

### 3.2. Apparatus and Procedures

Surface tension measurements were carried out by a model JYW-200B surface tensiometer (Chengde Dahua Testing Instrument Co., Ltd., Chengde, Hebei, China). The temperature was controlled with the help of a thermostatic bath. The surface tension was measured through a single-measurement method and all tests were repeated at least twice until the results were repeatable.

## 4. Conclusions

In summary, the aggregation behavior of long-chain piperidinium ILs C_n_PDB (*n* = 12, 14, 16) in EAN were investigated in this work. Through surface tension measurements, the *cmc*, γ*_cmc_* and Π*_cmc_* can be obtained and thermodynamic parameters related to the micellization can be calculated. Through an investigation of the effect of the alkyl chain length of the ILs, it can be concluded that the longer the alkyl chain the better characteristics the ILs possess. By analyzing the thermodynamic parameters at different temperatures, it can be established that the C_n_PDB micelle formation is an entropy-driven process. The DPD simulation clearly showed the micellization process of C_n_PDB in EAN. We expect our work will help better understand the aggregation behavior ILs in EAN.

## References

[1] Plechkova N.V., Seddon K.R. (2008). Applications of ionic liquids in the chemical industry. Chem. Soc. Rev..

[2] Bates E.D., Mayton R.D., Ntai I., Davis J.H. (2002). CO_2_ capture by a task-specific ionic liquid. J. Am. Chem. Soc..

[3] Wasserscheid P., Keim W. (2000). Ionic liquids-new solutions for transition metal catalysis. Angew. Chem. Int. Ed..

[4] Wasserscheid P. (2006). Chemistry: Volatile times for ionic liquids. Nature.

[5] Dupont J., de Souza R.F., Suarez P.A. (2002). Ionic liquid (molten salt) phase organometallic catalysis. Chem. Rev..

[6] Fletcher K.A., Pandey S. (2004). Surfactant aggregation within room-temperature ionic liquid 1-ethyl-3-methylimidazolium bis (trifluoromethylsulfonyl) imide. Langmuir.

[7] Cole-Hamilton D.J. (2003). Homogeneous catalysis—New approaches to catalyst separation, recovery, and recycling. Science.

[8] Wang X., Liu J., Yu L., Jiao J., Wang R., Sun L. (2013). Surface adsorption and micelle formation of imidazolium-based zwitterionic surface active ionic liquids in aqueous solution. J. Colloid Interface Sci..

[9] Wang H., Zhang L., Wang J., Li Z., Zhang S. (2013). The first evidence for unilamellar vesicle formation of ionic liquids in aqueous solutions. Chem. Commun..

[10] Jiao J., Zhang Y., Fang L., Yu L., Sun L., Wang R., Cheng N. (2013). Electrolyte effect on the aggregation behavior of 1-butyl-3-methylimidazolium dodecylsulfate in aqueous solution. J. Colloid Interface Sci..

[11] Cheng N., Yu P., Wang T., Sheng X., Bi Y., Gong Y., Yu L. (2014). Self-Aggregation of New Alkylcarboxylate-Based Anionic Surface Active Ionic Liquids: Experimental and Theoretical Investigations. J. Phys. Chem. B.

[12] Cheng N., Hu Q., Bi Y., Xu W., Gong Y., Yu L. (2014). Gels and Lyotropic Liquid Crystals: Using an Imidazolium-Based Catanionic Surfactant in Binary Solvents. Langmuir.

[13] Rogers R.D., Seddon K.R. (2003). Ionic liquids—Solvents of the future. Science.

[14] Welton T. (1999). Room-temperature ionic liquids. Solvents for synthesis and catalysis. Chem. Rev..

[15] Liu J., Zhao M., Zhang Q., Sun D., Wei X., Zheng L. (2011). Interaction between two homologues of cationic surface active ionic liquids and the PEO-PPO-PEO triblock copolymers in aqueous solutions. Colloid Polym. Sci..

[16] Song C.E. (2004). Enantioselective chemo-and bio-catalysis in ionic liquids. Chem. Commun..

[17] Zhao D., Wu M., Kou Y., Min E. (2002). Ionic liquids: Applications in catalysis. Catal. Today.

[18] Wang Y., Yang H. (2005). Synthesis of CoPt nanorods in ionic liquids. J. Am. Chem. Soc..

[19] Bittner B., Wrobel R.J., Milchert E. (2012). Physical properties of pyridinium ionic liquids. J. Chem. Thermodyn..

[20] Domańska U., Królikowski M., Ramjugernath D., Letcher T.M., Tumba K. (2010). Phase equilibria and modeling of pyridinium-based ionic liquid solutions. J. Phys. Chem. B.

[21] Wang N.N., Zhang Q.G., Wu F.G., Li Q.Z., Yu Z.W. (2010). Hydrogen bonding interactions between a representative pyridinium-based ionic liquid [BuPy] [BF_4_] and water/dimethyl sulfoxide. J. Phys. Chem. B.

[22] Neve F., Francescangeli O., Crispini A., Charmant J. (2001). A_2_[MX_4_] copper (II) pyridinium salts. From ionic liquids to layered solids to liquid crystals. Chem. Mater..

[23] Zhu X., Cui P., Zhang D., Liu C. (2011). Theoretical study for pyridinium-based ionic liquid 1-ethylpyridinium trifluoroacetate: Synthesis mechanism, electronic structure, and catalytic reactivity. J. Phys. Chem. A.

[24] Embs J.P., Burankova T., Reichert E., Hempelmann R. (2012). Cation dynamics in the pyridinium based ionic liquid 1-*N*-butylpyridinium bis ((trifluoromethyl) sulfonyl) as seen by quasielastic neutron scattering. J. Phys. Chem. B.

[25] Zeinolabedin Hezave A., Dorostkar S., Ayatollahi S., Nabipour S., Hemmateenejad B. (2013). Effect of different families (imidazolium and pyridinium) of ionic liquids-based surfactants on interfacial tension of water/crude oil system. Fluid Phase Equilib..

[26] Sastry N.V., Vaghela N.M., Macwan P.M., Soni S.S., Aswal V.K., Gibaud A. (2012). Aggregation behavior of pyridinium based ionic liquids in water—Surface tension, ^1^H NMR chemical shifts, SANS and SAXS measurements. J. Colloid Interface Sci..

[27] Harustiak M., Hronec M., Ilavsky J., Witek S. (1998). Micellar catalysts in the CoBr_2_ catalyzed oxidation of p-xylene in water. Catal. Lett..

[28] Sakaebe H., Matsumoto H. (2003). *N*-Methyl-*N*-propylpiperidinium bis(trifluoromethanesulfonyl)imide (PP13–TFSI)–novel electrolyte base for Li battery. Electrochem. Commun..

[29] Lethesh K.C., van Hecke K., van Meervelt L., Nockemann P., Kirchner B., Zahn S., Binnemans K. (2011). Nitrile-functionalized pyridinium, pyrrolidinium, and piperidinium ionic liquids. J. Phys. Chem. B.

[30] Matsumoto K., Hagiwara R., Ito Y. (2004). Room-temperature ionic liquids with high conductivities and wide electrochemical windows *N*-Alkyl-*N*-methylpyrrolidinium and *N*-Alkyl-*N*-methylpiperidinium fluorohydrogenates. Electrochem. Solid-State Lett..

[31] Zhao Y., Yue X., Wang X., Chen X. (2013). Lyotropic liquid crystalline phases with a series of *N*-alkyl-*N*-methylpiperidinium bromides and water. J. Colloid Interface Sci..

[32] Milioto S., Causi S., de Lisi R. (1993). Thermodynamic properties of some *N*-alkyl-*N*-methylpiperidinium chlorides and *N*-alkylpiperidine hydrochlorides in water. J. Solut. Chem..

[33] Zhao Y., Yue X., Wang X., Huang D., Chen X. (2012). Micelle formation by *N*-alkyl-*N*-methylpiperidinium bromide ionic liquids in aqueous solution. Colloids Surf. A.

[34] Walden P. (1914). Molecular weight and electrical conductivity of several fused salts. Bull. Russ. Acad. Sci..

[35] Weingärtner H., Knocks A., Schrader W., Kaatze U. (2001). Dielectric spectroscopy of the room temperature molten salt ethylammonium nitrate. J. Phys. Chem. A.

[36] Garlitz J.A., Summers C.A., Flowers R.A., Borgstahl G.E. (1999). Ethylammonium nitrate: A protein crystallization reagent. Acta Crystallogr. Sect. D.

[37] Summers C.A., Flowers R.A. (2000). Protein renaturation by the liquid organic salt ethylammonium nitrate. Protein Sci..

[38] Evans D.F., Yamauchi A., Roman R., Casassa E.Z. (1982). Micelle formation in ethylammonium nitrate, a low-melting fused salt. J. Colloid Interface Sci..

[39] Evans D.F., Yamauchi A., Wei G.J., Bloomfield V.A. (1983). Micelle size in ethylammonium nitrate as determined by classical and quasi-elastic light scattering. J. Phys. Chem..

[40] Tamura-Lis W., Lis L.J., Quinn P.J. (1987). Structures and mechanisms of lipid phase transitions in nonaqueous media: Dipalmitoylphosphatidylcholine in fused salt. J. Phys. Chem..

[41] Zhao M.W., Gao Y.A., Zheng L.Q. (2010). Liquid crystalline phases of the amphiphilic ionic liquid *N*-hexadecyl-*N*-methylpyrrolidinium bromide formed in the ionic liquid ethylammonium nitrate and in water. J. Phys. Chem. B.

[42] Evans D.F., Kaler E.W., Benton W.J. (1983). Liquid crystals in a fused salt: Beta, gamma distearoylphosphatidylcholine in *N*-ethylammonium nitrate. J. Phys. Chem..

[43] Araos M.U., Warr G.G. (2005). Self-assembly of nonionic surfactants into lyotropic liquid crystals in ethylammonium nitrate, a room-temperature ionic liquid. J. Phys. Chem. B.

[44] Evans D.F., Chen S.H., Schriver G.W., Arnett E.M. (1981). Thermodynamics of solution of nonpolar gases in a fused salt. Hydrophobic bonding behavior in a nonaqueous system. J. Am. Chem. Soc..

[45] Kang W., Dong B., Gao Y., Zheng L. (2010). Aggregation behavior of long-chain imidazolium ionic liquids in ethylammonium nitrate. Colloid Polym. Sci..

[46] Shi L., Zhao M., Zheng L. (2011). Micelle formation by *N*-alkyl-*N*-methylpyrrolidinium bromide in ethylammonium nitrate. Colloids Surf. A.

[47] Greaves T.L., Weerawardena A., Fong C., Drummond C.J. (2007). Many protic ionic liquids mediate hydrocarbon-solvent interactions and promote amphiphile self-assembly. Langmuir.

[48] Greaves T.L., Weerawardena A., Fong C., Drummond C.J. (2007). Formation of amphiphile self-assembly phases in protic ionic liquids. J. Phys. Chem. B.

[49] Thomaier S., Kunz W. (2007). Aggregates in mixtures of ionic liquids. J. Mol. Liquids.

[50] Li N., Zhang S.H., Zheng L.Q., Dong B., Li X.W., Yu L. (2008). Aggregation behavior of long-chain ionic liquids in an ionic liquid. Phys. Chem. Chem. Phys..

[51] Blesic M., Lopes A., Melo E., Petrovski Z., Plechkova N.V., Canongia Lopes J.N., Rebelo L.P.N. (2008). On the self-aggregation and fluorescence quenching aptitude of surfactant ionic liquids. J. Phys. Chem. B.

[52] Zana R. (2002). Dimeric (gemini) surfactants: Effect of the spacer group on the association behavior in aqueous solution. J. Colloid Interface Sci..

[53] Jaycock M.J., Parfitt G.D. (1981). Chemistry of Interfaces.

[54] Yang C., Chen X., Qiu H., Zhuang W., Chai Y., Hao J. (2006). Dissipative particle dynamics simulation of phase behavior of aerosol OT/water system. J. Phys. Chem. B.

[55] Lava K., Binnemans K., Cardinaels T. (2009). Piperidinium, piperazinium and morpholinium ionic liquid crystals. J. Phys. Chem. B.

